# Enhanced angiogenesis by the hyaluronic acid hydrogels immobilized with a VEGF mimetic peptide in a traumatic brain injury model in rats

**DOI:** 10.1093/rb/rbz027

**Published:** 2019-08-05

**Authors:** Jiaju Lu, Fengyi Guan, Fuzhai Cui, Xiaodan Sun, Lingyun Zhao, Ying Wang, Xiumei Wang

**Affiliations:** 1 State Key Laboratory of New Ceramics and Fine Processing, Key Laboratory of Advanced Materials of Ministry of Education, School of Materials Science and Engineering, Tsinghua University, Beijing 100084, China; 2 Beijing Center of Neutral Regeneration and Repair, Key Laboratory for Neurodegenerative Disease of the Ministry of Education, Capital Medical University, Beijing 100069, China

**Keywords:** traumatic brain injury, hyaluronic acid, angiogenesis, VEGF mimetic peptide

## Abstract

Angiogenesis plays an important role in brain injury repair, which contributes to the reconstruction of regenerative neurovascular niche for promoting axonal regeneration in the lesion area. As a major component of developing brain extracellular matrix, hyaluronic acid (HA) has attracted more attention as a supporting matrix for brain repair. In the present study, HA-KLT hydrogel was developed via modifying HA with a VEGF mimetic peptide of KLT (KLTWQELYQLKYKGI). The characterization of the hydrogel shows that it could provide a porous, three-dimensional scaffold structure, which has a large specific surface area available for cell adhesion and interaction. Compared with the unmodified HA hydrogel, the HA-KLT hydrogel could effectively promote the attachment, spreading and proliferation of endothelial cells *in vitro*. Furthermore, the pro-angiogenic ability of hydrogels *in vivo* was evaluated by implanting them into the lesion cavities in the injured rat brain. Our results showed that the hydrogels could form a permissive interface with the host tissues at 4 weeks after implantation. Moreover, they could efficiently inhibit the formation of glial scars at the injured sites. The HA-KLT hydrogel could significantly increase the expression of endoglin/CD105 and promote the formation of blood vessels, suggesting that HA-KLT hydrogel promoted angiogenesis *in vivo*. Collectively, the HA-KLT hydrogel has the potential to repair brain defects by promoting angiogenesis and inhibiting the formation of glial-derived scar tissue.

## Introduction

Traumatic brain injury (TBI) is a major health and socioeconomic problem worldwide. Approximately 1.7 million people each year suffer TBI in the United States, resulting in a debilitating loss of sensory and motor function [1]. TBI leads to a complex inhibitory microenvironment, including ischemia, the proliferation of inflammatory cells, the formation of glial-derived scar tissue and so on [2, 3]. Recent studies show that angiogenesis plays an important role in brain repair, and it could modify the inhibitory microenvironment after TBI [4].

Angiogenesis is a highly regulated process that involves the formation of new blood vessels through sprouting from pre-existing vessels by activation, migration and proliferation of vascular endothelial cells (ECs) [5]. It is fundamental to brain development and repair. The coupling of angiogenesis and neurogenesis has been observed in a specialized microenvironment or ‘neurovascular unit’ in the adult brain [6, 7]. Newly formed blood vessels not only provide oxygen and nutrients to brain tissues but also contribute to neurogenesis and neuronal remodeling [8, 9]. Severe TBI can directly lead to vasculature damage, consequently, neuronal cell death and dysfunction would occur in the absence of an adequate blood supply. The formation of an ischemic environment is considered as secondary injury processes after TBI [10, 11]. Therefore, therapeutic strategies aiming to improve the regenerative microenvironment via enhancing angiogenesis may bring promising results for TBI.

Three-dimensional (3D) hydrogels could fill up the irregularly shaped lesion cavities in the injured brain, and provide a favorable microenvironment with trophic factor for cell growth and proliferation [12]. Therefore, various hydrogels have been applied as a supporting matrix for CNS repair, such as collagen [13], chitosan [14], synthetic polymer [15, 16] and self-assembling peptide [17]. Among these hydrogel scaffolds, hyaluronic acid (HA) has attracted more attention because it is an important component of the brain extracellular matrix (ECM) [18]. HA, also known as hyaluronan, is a naturally occurring linear glycosaminoglycan (GAG) consisting of repeating disaccharide units of β-(1,4)-d-glucuronic acid and β-(1,3)-*N*-acetyl-d-glucosamine [19]. It could bind with other components of ECM in the body, such as GAGs and proteoglycans, through specific HA–cell surface receptors interaction [20, 21]. Therefore, HA plays a vital role in cell proliferation and migration during a wide variety of biological processes, such as tissue regeneration and angiogenesis [22]. However, previous studies suggested that the crosslinked high-molecular-weight (HMW)-HA could inhibit angiogenesis [23], whereas degraded HA fragments of low molecular weight induce EC proliferation and migration. Therefore, angiogenic growth factors such as vascular endothelial growth factor (VEGF) were loaded to HA scaffolds to increase angiogenesis. Montesano *et al.* found that there was a synergistic interaction between VEGF and oligosaccharide HA on angiogenesis *in vitro* [24]. Peattie *et al.* applied a modified HMW-HA hydrogel for localized delivery of VEGF and basic fibroblast growth factor (bFGF) in the ear pinna of mice. The results showed that the growth factor pre-loaded HA hydrogel could induce new capillary vessel growth *in vivo* [23]. However, the clinical application of recombinant VEGF or other recombinant proteins faces many challenges, such as high preparation costs, a specific target tissue retention and unwanted side effects [25].

A promising alternative is to develop HA hydrogel modified with a functional peptide motif that could mimic the biological activity of growth factors. Peptides are much smaller than full-length growth factors, which are relatively easier to synthesize and cheaper [26]. In addition, peptides could only interact with specific receptors to avoid side effects due to their small size and structural stability, showing that they are safer than growth factors [27]. The functional motif KLT (KLTWQELYQLKYKGI) is a VEGF mimetic peptide. It was designed based on the VEGF helix sequence 17-25, which could activate VEGF receptors and promote angiogenesis [27, 28]. It has been demonstrated to promote EC growth, migration and tubulogenesis [29]. We previously developed a series of modified HA hydrogels by introducing RGD or IKVAV peptide sequence into HA backbones to increase cell attachment and nerve regeneration [30, 31]. In this study, we developed a HA-KLT hydrogel by immobilizing HA with VEGF peptide motif KLT in an attempt to promote angiogenesis for brain tissue engineering.

Here, the *in vitro* proangiogenic properties of HA hydrogel unmodified or modified with KLT motif were first studied. Furthermore, we implanted these hydrogel scaffolds in the rat brain injure model, and evaluated the effects on inducing angiogenesis and neural tissue formation *in vivo*.

## Materials and methods

### Materials

Sodium hyaluronate (HA-Na, molecular weight: 2.6–2.7 × 10^6^ Da, injectable grade) was purchased from Shandong Fureda Biochem (Fureda, Jinan, Shandong, China). Ethyl-*N*,*N*-dimethylaminopropyl carbodiimide (EDC) and adipic dihydrazide (ADH) were acquired from Sigma-Aldrich (St. Louis, MO, USA). 1,1′-carbonyldiimidazole (CDI) was purchased from J&K Chemical (Beijing, China). The KLT peptide (KLTWQELYQLKYKGI) was synthesized and purified by Chinese Peptides Co., Ltd. (Hangzhou, Zhejiang, China). Primary isolated human umbilical vein endothelial cells (HUVECs) were commercially obtained from cell culture center of Basic Medical Sciences, Chinese Academy of Medical Sciences. Dulbecco’s modified Eagle medium (DMEM) and fetal bovine serum (FBS) were obtained from Gibco (Invitrogen, CA, USA). Cell Counting Kit-8 (CCK-8) was purchased from Dojindo (Dojindo Laboratories, Kumamoto, Japan). Rhodamine-phalloidin and 4′,6-diamidino-2-phenylindole (DAPI) were acquired from Sigma-Aldrich (St. Louis, MO, USA). Sprague-Dawley (SD) female rats (8-week-old, 180–200 g) were purchased from Beijing Vital River Laboratory Animal Technology Co. Ltd. (Beijing, China). Goat antirat endoglin CD105 directed to the endoglin was purchased from Abcam (Cambridge, UK). Histostain-SP Kit was purchased from Zsbio (Beijing, China).

### Preparation of the crosslinked HA hydrogel

**Figure 1:**
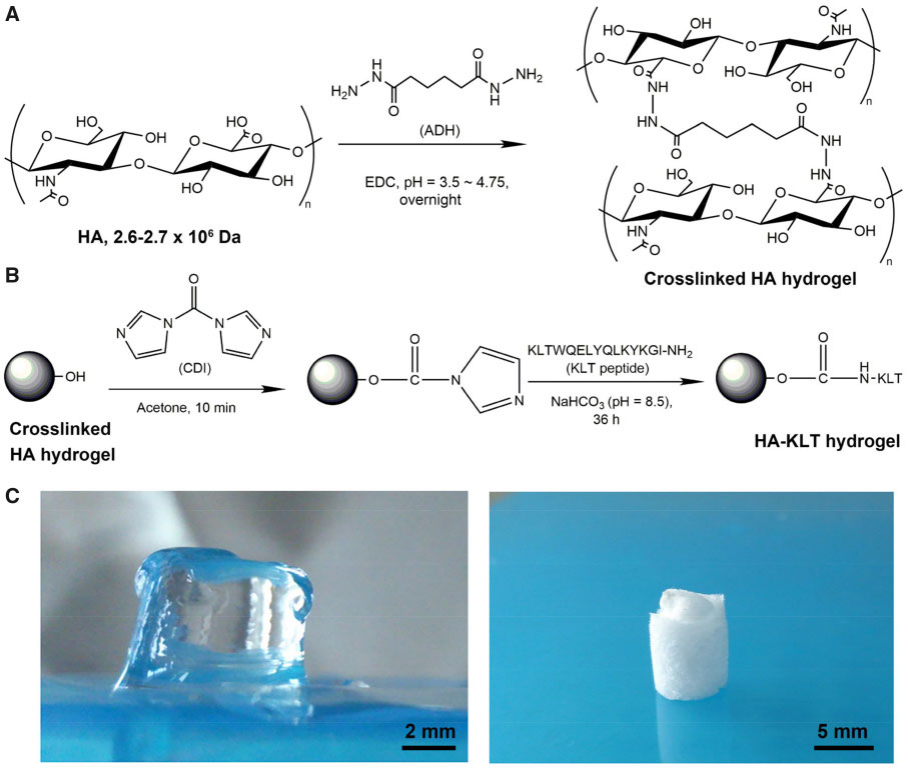
Schematic illustration of the formation of crosslinked HA hydrogel (**A**) and HA-KLT hydrogel via amide reaction (**B**). (**C**) The morphologies of wet HA-KLT hydrogel (left) and dry HA-KLT gel after freeze drying (right)

The preparation of the crosslinked HA gel followed the protocols previously reported, as shown in Fig. 1A [32]. Polylysine (PLL) was used to improve cell adhesion. Briefly, HA-Na was dissolved in distilled water to form a solution at a concentration of 10 mg/ml with thorough stirring at room temperature overnight, followed by adding the PLL at the ratio of 5:1 by weight (HA: PLL). The ADH (6 × the HA by weight) was then added. After stirring thoroughly, the pH of the mixed solution was adjusted to 3.5–4.75 by adding 0.1 N HCl and EDC was finally added to the mixture with thorough stirring. The mixture was then allowed to gelate at 4°C overnight. The hydrogels were washed thoroughly with deionized water eight times (10 min/per times) and then lyophilized.

### Preparation of the HA-KLT hydrogel

Activation of the crosslinked HA hydrogel and peptide immobilization was the protocols previously reported, as shown in Fig. 1B [16]. The crosslinked HA hydrogel was washed in acetone for several times. A CDI solution in dry acetone (20 mg/ml) was then added to the HA hydrogel with gentle stirring for 10 min. The HA hydrogel was washed with acetone five times with 5 ml to remove the extra CDI. The KLT peptide was dissolved in sterile 100 mM NaHCO_3_ (pH = 8.5) at the concentration of 5 mg/ml. The KLT peptide reacted with CDI activated HA hydrogel for 36 h with gentle shaking at room temperature. Then the immobilized HA-KLT hydrogel was washed thoroughly using 0.1 M PBS five times over a 48-h period and lyophilized. The re-swelled hydrogel for animal implantation was kept in sterile 0.1 M PBS at 4°C.

### Scanning electron microscopy

The HA and HA-KLT hydrogels were fixed in 2% glutaraldehyde solution for 2 h, dehydrated with a series of graded ethanol (30, 50, 70, 80, 90, 95 and 100%) and 2-methyl-2-propanol solutions (50, 75, 100%). Once dehydrated, samples were freeze dried overnight. The samples were sputter coated with 10 nm gold and examined using a JEOL JSM-7001F field emission gun scanning electron microscope (FEG-SEM) at 10 kV.

### Fourier transform infrared spectroscopy

Fourier transform infrared (FTIR) measurements were performed for HA powder, dry HA gel and dry HA-KLT gel using a Nicolet 6700 spectrophotometer (Nicolet Instrument Co., USA). The FTIR spectrum of all samples were recorded to be in the range of 400–4000 cm^−1^ with a resolution of 4 cm^−1^.

### Rheology

Rheological tests were performed as previously reported [33]. The lyophilized sample sponges were prepared with PBS solutions to form the hydrated hydrogels at swelling equilibrium. The gels (15 mm diameter disk, 1.3 mm thick) were measured with a MCR300 constant stress rheometer (Paar Physica, Austria) in the parallel-plate geometry with a variable gap. The samples were kept in water at a controlled temperature (37°C) and tested in a dynamic regime at small amplitudes of deformation (1%). To determine the variation of storage moduli (*G*′) and loss moduli (*G*″), the small-amplitude oscillatory shear measurements were recorded. The intervals were varying from 0.9 to 1.3 mm and the frequencies were ranging from 1 to 100 rad/s under various compressions levels. The linear viscosity region was determined by stress sweep measurements.

### Cell culture

HUVECs were cultured with DMEM supplemented with 10% FBS, 10 ng/ml endothelial cell growth factor (EGF) and 1% penicillin‐streptomycin antibiotic solutions. Cells were maintained in a humidified atmosphere of 5% CO_2_ at 37°C. Culture media was changed every 3 days. Cell passaging was done once cells reached 70–80% confluency using the 0.25% trypsin enzymatic digestion method. Passage 3-8 cells were used in this study for two-dimensional (2D) culture studies.

### Cell spreading

The samples (6 mm × 6 mm × 3 mm, *n* = 6) were sterilized with ^60^Co-γ radial with an absorbable dose of 15 kGy. Subsequently, the samples were placed into 48-well culture plates, and 500 μl of cell suspensions were seeded onto the surface of each hydrogel at 8 × 10^4^ cell/ml of cell-seeding density. After seeding, the cell/gel constructs were incubated in a humidified atmosphere of 5% CO_2_ at 37°C. Culture media was changed every 3 days. The cell/gel constructs were harvested at 2, 4 and 6 days. The samples were fixed, dehydrated and dried for SEM analysis. In addition, the HUVECs on the gels were stained with rhodamine phalloidin and DAPI according to the manufacturer’s instruction. Briefly, cell/gel constructs were washed three times with 0.01 M PBS solution. Then they were fixed with 4% formaldehyde for 20 min. After being washed by PBS, the samples were permeabilized in 0.1% Triton-X100 solution for 10 min and blocked with 1% BSA for 30 min, followed by staining with rhodamine-phalloidin for 40 min and DAPI for 20 min. After washing, cells were observed with a laser scanning confocal microscope (LSCM, LSM 710, Carl Zeiss Microimaging GmbH, Jena, Germany).

### Cell proliferation

The samples (6 mm × 6 mm × 3 mm, *n* = 3) were placed into 48-well culture plates, and 100 μl of cell suspensions at a density of 1 × 10^5^ cells/ml were inoculated onto the surface of each hydrogel. After cell seeding, the cell/gel constructs were incubated in a humidified atmosphere of 5% CO_2_ at 37°C. Culture medium was replaced with fresh medium every day. Cell numbers at 3, 5 and 7 days were counted using a CCK-8 solution.

### Animals and surgical procedures

A total of 23 adult male SD rats weighing approximately 180–220 g were randomly allocated into three groups: HA group (*n* = 10), HA-KLT group (*n* = 10) and blank control group (*n* = 3). Animal procedures were performed according to Guides for the Care and Use of Laboratory Animals from the Chinese Ministry of Public Health and United States National Institutes of Health. The following surgical protocol for traumatic brain tissue injury was modified from the previous literature [34]. Briefly, animals were anesthetized with the intraperitoneal injection of 25 mg/kg of 3% pentobarbital sodium, and then positioned on a stereotactic apparatus. The head and the neck were shaved and thoroughly cleaned. Under aseptic conditions, a midline incision was made and a bone flap of about 3 × 5 mm^2^ on the left of the midline was removed. The dura was incised and peeled away in order to expose the parietal cortex. A block of cortical tissue (5 × 3 × 3 mm^3^) was removed and a lesion cavity was made in the left frontal cortex region. A piece of HA or HA-KLT hydrogel sized to the dimensions and shape of the cavity was placed into the lesion site. The animals of the blank group received no implant and were considered as a control group. The bleeding was controlled with thrombin-soaked gel foam. The bone flap was put back and the scalp sutured to close the wound. The rats were given antibiotics (ampiciline 200–400 mg/kg) at the end of the surgical procedure.

### Histology assay

Brain tissues from HA and HA-KLT groups were harvested at 4 weeks after the procedures (*n* = 5). Before sacrifice, all animals were perfused through the heart with a 0.9% saline solution and 4% paraformaldehyde under deep anesthesia. The brains were taken out and postfixed for the following histologic assay. Frozen sections with 40-μm thickness were cut by freezing microtome and prepared for the staining. All the sections were processed for hematoxylin and eosin (H&E) staining and endoglin/CD105 immunohistochemical staining.

### Western blot

Brain tissues were harvested at 4 weeks after the procedures (*n* = 5) and homogenized in 0.1% sodium dodecylsulfate (SDS)-RIPA lysis buffer supplemented with protease and phosphatase inhibitors. Total lysates were collected and equal quantities of protein resolved by SDS–polyacrylamide gel electrophoresis and the immunoblotting. Primary antibodies used were anti-endoglin/CD105 monoclonal antibody (mAb) and anti-β-actin mAb, followed by incubating with HRP-conjugated secondary antibody. Detection was performed with the Li-Cor Odyssey imaging system and quantitated with Image J software.

### Statistical analysis

All quantitative results were reported as mean ± standard deviation. Student’s *t*-test was used to compare the differences using SPSS software. A value of *P *<* *0.05 was considered statistically significant.

## Results

### Preparation of the crosslinked HA and HA-KLT hydrogel

The preparation scheme of the crosslinked HA hydrogel is shown in Fig. 1A. The surface carboxyl groups of HA were reacted with ADH by adjusting pH to 3.5–4.75. Because the hydrazide moieties of ADH are nucleophilic at this pH, which could contribute to the efficient coupling of the carboxylic acid groups of HA with the hydrazide of ADH. In the final step, the hydrogel was formed by using EDC as the cross-linking agent. The HA-KLT hydrogel was prepared by the coupling reaction of the amino groups of KLT peptide to the carboxylic acid groups of HA using CDI activation, which was stable and highly biocompatible (Fig. 1B). Figure 1C showed the typical morphologies of HA-KLT hydrogel.

**Figure 2:**
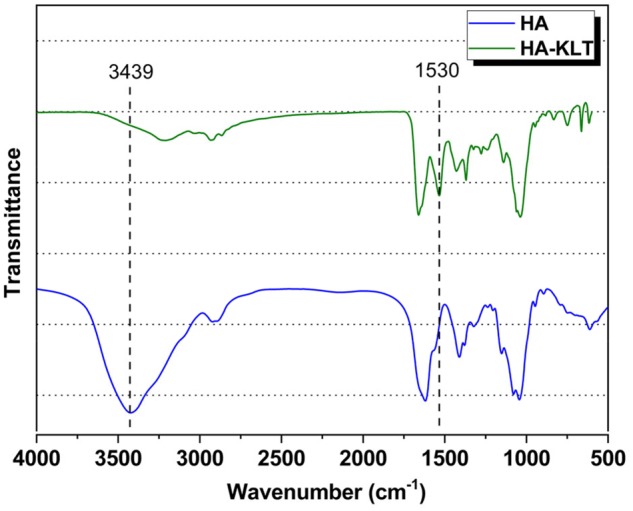
FTIR spectra of HA and HA-KLT hydrogel

To determine the chemical modification of the HA during the crosslinking reaction, the HA powder and HA-KLT gel were analysed with FTIR as shown in Fig. 2. The reduction in the intensity of the stretch vibration at 3439 cm^−1^ originated from the benzene ring structure of KLT peptide, indicating that KLT peptide was introduced into HA. In addition, the reaction of HA with KLT peptide resulted in an increase in the intensity of the stretch vibration of C = O at 1530 cm^−1^, indicating the formation of an amide between HA and KLT peptide. The result of the FTIR analysis revealed that modification was successful.

### Characterization of HA-KLT hydrogel

**Figure 3:**
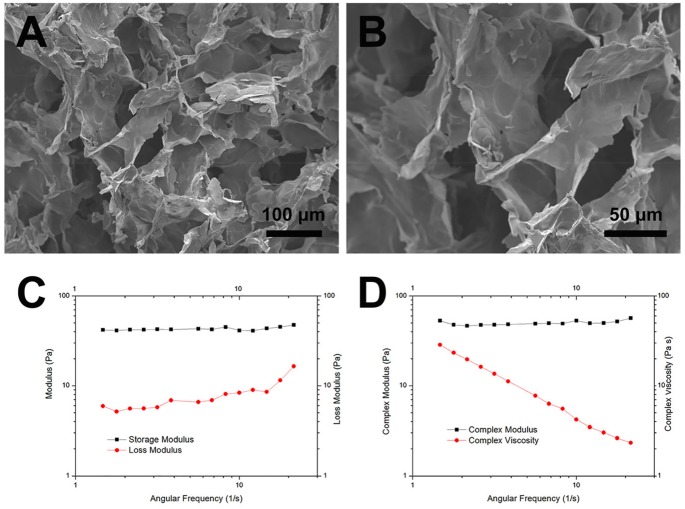
Ultrastructure and rheological evaluation of HA-KLT hydrogels. (**A**) SEM image of the HA-KLT hydrogel structure. (**B**) Higher magnification of image of (A) revealed the interior morphology of HA-KLT hydrogel. (**C**) Storage modulus (*G*′) and loss modulus (*G*″), as function of angular frequency for the HA-KLT hydrogel at 37°C. (**D**) Complex modulus (G*) and complex viscosity (η*) of the HA-KLT hydrogel at 37°C

The microstructure of HA-KLT hydrogel after freeze drying was observed by SEM. As shown in Fig. 3A and B, the HA-KLT gel presented a heterogeneous, continuous interconnected porous structure with pore diameters in the range of 50–200 μm. Such a porous structure of HA-KLT hydrogel may not only provide enough space for migration of ECs and neural cells but also support the formation of blood vessels. Pore size is an important parameter of 3D porous hydrogel scaffold, which affects the attachment and spreading of cells [35]. It has been suggested that 3D scaffolds with a distribution of pores (50–160 μm) are optimal for smooth muscle ECs and nerve cells growth [36]. In addition, the pore size of scaffolds should be larger than 50–100 μm, which could enable blood vessel ingrowth and cell migration [37].

To investigate the mechanical properties and gelation conditions of HA-KLT hydrogel, rheological measurements were performed. Frequency sweep dynamic rheology data of HA-KLT hydrogel were shown in Fig. 3C. The storage modulus (*G*′) and loss modulus (*G*″) represented the energy stored or dissipated per unit strain respectively. The HA-KLT hydrogel exhibited a solid-like behavior, which showed that its *G*′ was more than *G*″ during the measuring process. In addition, oscillatory tests were further conducted to determine the complex modulus (G*) and complex viscosity (η*) of the HA-KLT hydrogel. As shown in Fig. 3D, a constant G* and an η* that increased with decreasing frequency were observed in the HA-KLT gel. The values were similar to those previously determined for the behavior of the rat brain tissue [38].

These results showed that the HA-KLT hydrogel has a highly porous structure, which also has a large specific surface area for cell growth and migration. In addition, the HA-KLT hydrogel matched the mechanical properties of the rat brain cortex, so that it could fill into the cavities of injured brains and support tissue regeneration.

### Cell adhesion, spreading and proliferation

**Figure 4:**
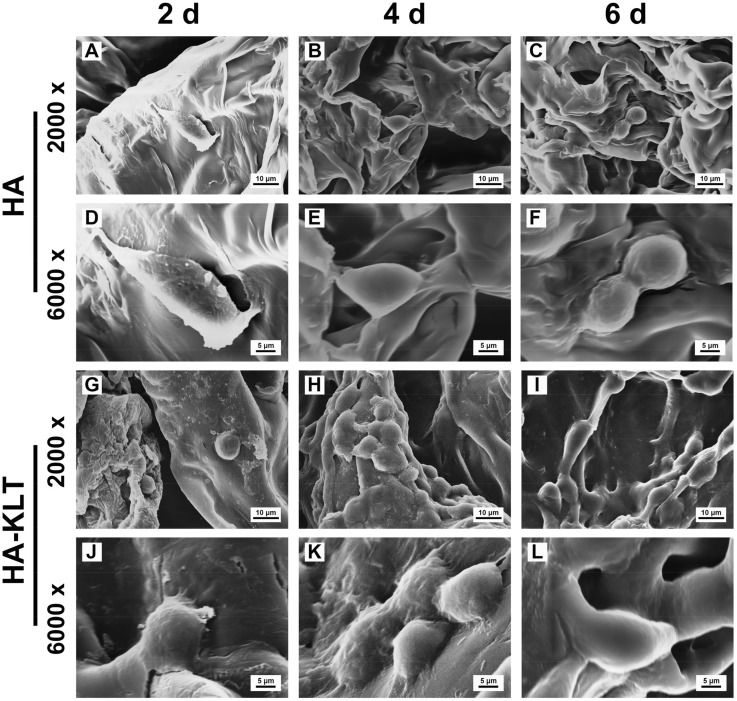
SEM images showed the interaction between HUVECs and hydrogel of HA or HA-KLT. (**A**, **B** and **C**) HUVECs cultured on HA hydrogel at 2, 4 and 6 days, respectively. Higher magnification of image of (A, B and C) are shown in the (**D**, **E** and **F**), respectively. (**G**, **H** and **I**) HUVECs cultured on HA-KLT hydrogel at 2, 4 and 6 days, respectively. Higher magnification of image of (G, H and I) are shown in **J**, **K** and **L**, respectively

**Figure 5:**
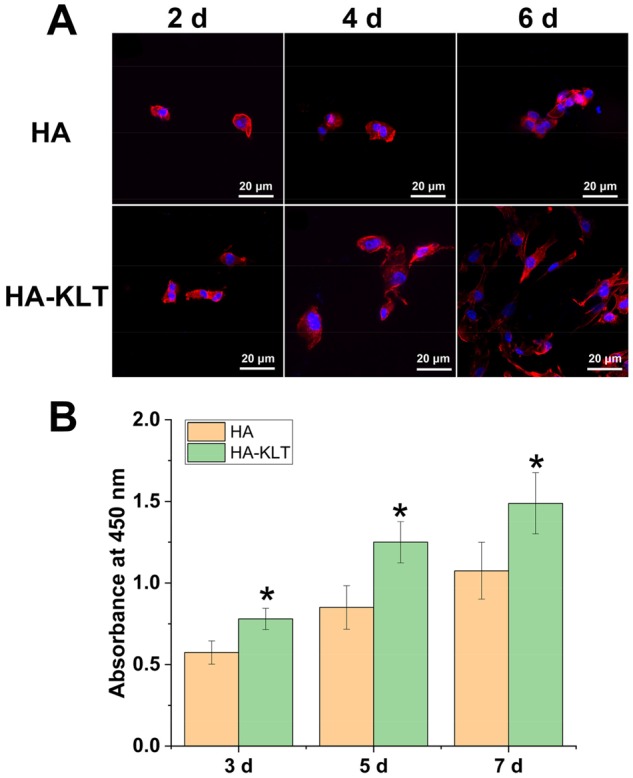
Adhesion and proliferation of HUVECs on HA and HA-KLT gels. (**A**) LSCM image of HUVECs on the HA and HA-KLT hydrogel at 2, 4 and 6 days. Cells were stained with rhodamine phalloidin for actin and DAPI for nucleus. (**B**) Cell proliferation on the HA and HA-KLT gels by CCK8 assay after 3, 5 and 7 days. Data are expressed as the mean ± standard deviation for *n* = 3. *P <* *0.05

To evaluate the function of the HA and HA-KLT hydrogel scaffolds in supporting HUVECs *in vitro*, the adhesion and spreading behaviors of HUVECs on the surface of the HA and HA-KLT hydrogel were investigated by SEM and LSCM. As shown in Fig. 4, the initial cell attachments on the HA and HA-KLT gel were similar, and the cells displayed the spherical morphology in both HA and HA-KLT groups after 2-day culture. However, the cellular adhesion on the HA-KLT gel was effectively improved as compared with that of the HA gel after 4 and 6 days of cell culture. We observed that a large number of adherent cells grew along the pore wall of the HA-KLT hydrogel, suggesting that HUVECs had extended into the inner structure of the hydrogel. In the HA gel scaffold, the migration of HUVECs could be rarely observed. The LSCM images also clearly showed that the adhesion of HUVECs occurred preferentially for the HA-KLT gel (Fig. 5A). The cells on the HA-KLT gel presented a larger cell-spreading area than that of the HA gel after 4-day culture. In contrast, the cells on the HA gel remained the round shaped.

Proliferations of HUVECs on the HA and HA-KLT gels were further evaluated by CCK-8 assay at 3, 5 and 7 days (Fig. 5B). The number of cells increased with time, suggesting that the cells proliferated well on both HA and HA-KLT groups. However, the number of cells on the HA-KLT group was significantly higher than that of the HA group at all days of cell culture (*P *<* *0.05). These results demonstrated that the HA-KLT group modified with the VEGF mimetic motif could promote HUVEC adhesion, spreading and proliferation.

### Histological analysis

**Figure 6:**
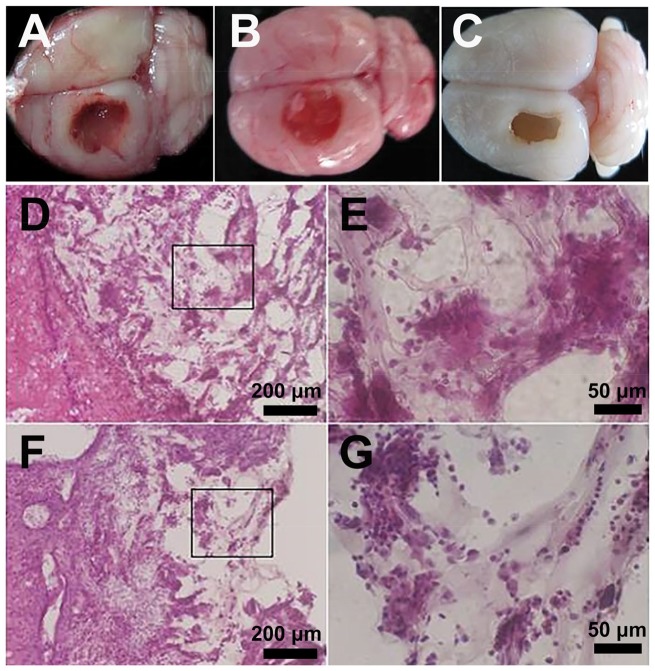
In the left frontal cortex region of rats, lesion cavities (5 × 3 × 3 mm^3^) were mechanically created. The HA or HA-KLT gel was injected immediately to fill up the lesion cavities. The harvested brain tissue of HA group (**A**), HA-KLT group (**B**) and blank control group (**C**) after implantation for 4 weeks. Representative H&E staining of tissues embedded with HA gel were shown in (**D**) 10× and (**E**) 40× (indicated in black box) views. H&E staining of tissues embedded with HA-KLT gel were shown in (**F**) 10× and (**G**) 40× (indicated in black box) views

To test the biocompatibility and bioactivity of the HA-KLT hydrogel, we used a traumatic rat brain model. In the left frontal cortex region of the rat, a lesion cavity (5 × 3 × 3 mm^3^) was mechanically created. The hydrogel scaffold was immediately filled into the damaged area after injury. At 4 weeks after surgery, the brain tissues from the HA, HA-KLT and blank control group were harvested. From the morphological examinations, the hydrogel scaffolds filled the wound defects well (Fig. 6A and B). The border of gel was closely connected with the brain tissue, and had been completely or partially merged. In addition, no macroscopic signs of inflammation or toxicity were evident at the injured site in the HA or HA-KLT group. However, the injured area in the blank control group still remained like a cavity, suggesting severe tissue loss and failed repair (Fig. 6C).

Histological analysis of brain sections by H&E staining was conducted at week 4. As shown in Fig. 6D–G, the border between the hydrogel and surrounding tissues was blurry in both HA and HA-KLT groups, suggesting that hydrogels could be degraded and the regenerative tissue could migrate into the hydrogels. In addition, fibrous capsules were not observed in these two groups. These results showed that the HA and HA-KLT hydrogel showed good biocompatibility *in vivo*, which could prevent the formation of glial scars.

### The influence of hydrogel on angiogenesis

**Figure 7:**
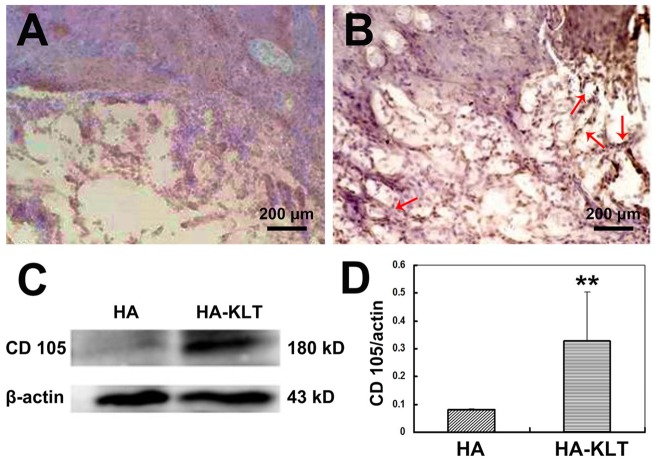
Immunohistochemical staining of endoglin/CD105 of the harvested brain tissue in the HA group (**A**) or HA-KLT group (**B**) after implantation for 4 weeks (red arrows indicate the formation of blood vessels). The expression of CD105 was further examined by western blotting (**C** and **D**). β-actin was used a reference. Data are expressed as the mean ± standard deviation for *n* = 3. **P <* *0.01

To assess the angiogenic effect of hydrogel for brain tissues, immunohistochemical staining of endoglin/CD105 was performed after 4 weeks of hydrogel implantation. Endoglin (CD105) is a homodimeric transmembrane glycoprotein, which is used as a specific marker of neoformed vessels and is mainly expressed by ECs in both rat and human brain [39]. In the human brain, endoglin is expressed in the endothelium of all vessels [40]. As shown in Fig. 7B, the HA-KLT hydrogel merged with host brain tissues, and large numbers of apparently formed new microvessels were distributed in the hydrogel scaffold, showing that neovascularization was improved by the HA-KLT gel. However, the number of blood vessels of the HA hydrogel was less than that of the HA-KLT hydrogel. In addition, the vessels in the HA group often did not show a complete endothelial border (Fig. 7A).

Figure 7C showed the results of western blot analysis of endoglin/CD105. Endoglin/CD105 was seen at 180 kDa of the HA-KLT group but little in the HA group. The endoglin/CD105 and β-actin bands were quantified by densitometry. The expression of endoglin/CD105 in the HA-KLT group was significantly higher than that in the HA group (Fig. 7D, *P *<* *0.01). These results suggested that the HA-KLT hydrogel could be active and recruit vascular ECs, and promote efficiently the formation of blood vessels in the brain tissue.

## Discussion

In the present study, we investigated whether HA modified with VEGF mimetic peptide motif KLT could promote angiogenesis and inhibit the formation of glial scars after TBI. Recently, the importance of vascularization in CNS repair is just emerging, especially for repairing ischemic brain injury [41]. Morgan *et al.* found a substantial increase in angiogenesis in an acceleration impact model of TBI, providing evidence for both angiogenesis and vasculogenesis following injury [42]. Newly formed blood vessels formed by ECs not only provide nutritional support but also play an important role in modulating the self-renewal and neurogenesis of neural stem cells [7]. Therefore, appropriate biomaterials with pro-angiogenic factors are essential for the reconstruction of the regenerative microenvironment in injured brain tissues.

HA is a major ECM component in the fetal mammalian brain, which plays an important role in tissue repair. Due to its biocompatibility, nonimmunogenicity and biodegradability, HA has been widely used in nerve tissue engineering [43]. Native HA is water soluble and cannot form a 3D hydrogel scaffold for cell adhesion and regrowth [44]. Functionalization of HA-based hydrogel scaffold has been obtained using various techniques [45]. In addition, the mechanical properties and degradation behaviors of HA hydrogel could be easily modified by changing the molecular weight of HA, crosslink density of HA or crosslinking agents in order to match the properties of native tissues [46–48]. In the present study, HA-based hydrogel was formed by a cross-linking reaction under mild condition. Furthermore, we modified HA hydrogel with the KLT peptide motif via a facile synthetic route. The HA-KLT hydrogel provides a porous, 3D scaffold structure, which mimics the structure and components of the ECM of brain tissues. The SEM images showed that HA-KLT hydrogel has a large specific surface area available for cell attachment and spreading (Fig. 3). After TBI, a glial scar forms in the injured brain tissues, which prevents axon regeneration and extension. In the rat, a dense fibrous scar surrounded by a glial membrane is constructed within the wound after 5–8 days, and the glial scar is fully formed by 8–14 days [49]. It is reported that HA could inhibit the formation of glial scar tissues via decreasing the thickness of gliosis and reducing the number of glial cells [50]. Our results showed that no astrocyte scar was found in the HA and HA-KLT hydrogel at 4 weeks after implantation, according to the HE staining and morphological data (Fig. 6). Both HA and HA-KLT hydrogels could reform the injured tissue structure and formed a permissive interface with the host tissue. In addition, HA-KLT hydrogel underwent degradation and was partially replaced by the newly formed brain tissues within 4 weeks (Fig. 6). According to our previous study, the crosslinked HA hydrogel with peptide sequences could be fully degraded after 12 weeks implantation *in vivo* [30].

Given the restorative effects of angiogenesis after brain tissue damage, numerous studies focused on the delivery of pro-angiogenic growth factors or chemokines to the injured site, such as VEGF [51], fibroblast growth factors (FGF) [52] and stromal cell-derived factor-1 (SDF-1) [53]. Among these pro-angiogenic growth factors and chemokines, VEGF is the most widely used one due to its specific mitogenic activity for vascular ECs. It could induce angiogenesis by binding and activating its receptors, resulting in the proliferation of newly formed blood vessels and elevation of vascular permeability [54]. After TBI, VEGF is a part of the molecular signaling network that mediates improved survival of *de novo* granule neurons [55]. Thau-Zuchman *et al.* explored the effect of continuous infusion of exogenous VEGF into the lateral ventricles of mice after TBI. Their results showed that VEGF not only significantly increased angiogenesis but also contributed to the proliferation and migration of neural cells via phospho-Akt signaling, suggesting its additional neuroprotective effects [51]. Therefore, VEGF has dual roles in nervous and vascular systems, which not only promotes angiogenesis but also plays an important role in neurogenesis and neuroprotection. The recombinant VEGF via intravenous administration has shown good potential for repairing TBI, but it could be rapidly degraded due to its low stability, which has a short half-life of only ∼30 min *in vivo* [56]. Although sustained delivery of VEGF could overcome its short lifetime through the physical or chemical attachment of VEGF to a scaffold, each approach is dependent on recombinant proteins [57, 58]. Therapeutic application of recombinant VEGF was found to induce side effects, such as local edema, inflammatory reactions and hypotension [59, 60]. In addition, the cost of recombinant VEGF is high due to its limited production resources. Therefore, we selected and synthesized the VEGF mimetic peptide sequence KLT. The KLT peptide has been shown to have a half-life of ∼5 h and degrade after 24 h in 50% human serum, which shows significant resistance to serum proteases due to its rigid and well folded peptide structure [61]. In addition, the bioactive peptide motifs have many advantages over growth factors, such as low price and safety. The *in vitro* results showed that the HA hydrogel modified with KLT motif could effectively promote the adhesion, spreading, proliferation of ECs, compared with the unmodified HA hydrogel (Figs 4 and 5). Moreover, the HA-KLT hydrogel could promote the formation of blood vessels and significantly increase the expression of endoglin/CD105 at 4 weeks after implantation, which showed that the HA-KLT hydrogel could improve angiogenesis compared with the unmodified HA hydrogel *in vivo* (Fig. 7).

In this study, we only focused on the effect of HA-KLT hydrogel on angiogenesis using a rat TBI model, and the functional recovery of injured animals would be further studied in the future work. Besides, it has been suggested that the delivery of a single pro-angiogenic factor maybe not enough to promote complete functional recovery and nerve regeneration. Therefore, regenerative strategies via HA-based hydrogel delivering simultaneously multiple biochemical cues will also be developed.

## Conclusions

In this study, we first developed the HA-KLT hydrogel by immobilizing HA with VEGF peptide motif for brain tissue engineering. HA-KLT hydrogel could provide a porous structure scaffold for cell adhesion and spreading. Compared with the HA group, the HA-KLT hydrogel contributed to the adhesion, spreading and proliferation of HUVECs *in vitro*. In addition, the HA-KLT hydrogel showed good biocompatibility *in vivo*. After implantation into a lesion of the brain tissues, the HA-KLT not only promoted angiogenesis but also inhibited the formation of glial scars at the injured site. Our study demonstrated that HA-KLT hydrogel may be a promising hydrogel scaffold for repairing TBI.

## Funding

This work was supported by the National Natural Science Foundation of China (31771056 and 81200931), the Tsinghua University Initiative Scientific Research Program (20161080091), the 111 Project (B17026) and a special fund from Key laboratory of Neurodegenerative diseases, Ministry of Education (PXM2019_026283_000002). 

*Conflict of interest statement*. None declared.
